# The 2034 FIFA World Cup in Saudi Arabia: A Catalyst for Global Health Innovation and Emergency Preparedness

**DOI:** 10.7759/cureus.90651

**Published:** 2025-08-21

**Authors:** Mobarak A Al Mulhim, Fadi Eissa, Amalia Voskanyan, Attila Hertelendy, Gregory Ciottone

**Affiliations:** 1 Department of Emergency Medicine, Beth Israel Deaconess Medical Center, Harvard Medical School, Boston, USA

**Keywords:** emergency preparedness, fifa world cup 2034, global health, telemedicine, vision 2030

## Abstract

The 2034 FIFA World Cup in Saudi Arabia offers a unique chance to advance global health and emergency preparedness for mass gatherings. Leveraging Vision 2030’s healthcare reforms, digital advancements (blockchain electronic health records, artificial intelligence (AI) diagnostics, telemedicine), and lessons from the 2024 Hajj’s heat challenges, Saudi Arabia aims to redefine medical readiness. This editorial advocates for innovative, equitable, and collaborative emergency medicine, integrating digital health, robust heat monitoring, and fair access to care. By pioneering MediTech innovations such as AI surveillance and autonomous medical delivery, the Kingdom can establish a new global standard for a health-first approach to mega-events.

## Editorial

The FIFA World Cup transcends its role as a global sporting spectacle; it serves as a critical stress test for a host nation’s capacity to safeguard the health and well-being of millions converging in a high-stakes, dynamic environment. As Saudi Arabia prepares to host the 2034 World Cup, the confluence of its ambitious national transformation plan, Vision 2030 [1], alongside evolving global health imperatives and the Kingdom’s distinctive geographic and climatic challenges, presents an unprecedented opportunity. This editorial posits that the 2034 World Cup can fundamentally redefine medical preparedness for mass gatherings, establishing a forward-looking blueprint for future global health security through focused emergency medicine strategies rooted in cutting-edge innovation, unwavering health equity, and robust cross-sector collaboration. This event offers a unique chance to demonstrate how a nation can leverage its strategic vision and lessons learned from previous large-scale events to build a resilient, technologically advanced, and patient-centric healthcare framework capable of managing diverse health risks.

At the core of Saudi Arabia’s readiness for such a monumental event lies Vision 2030, a comprehensive strategic framework launched in 2016. This national blueprint aims to diversify the Kingdom’s economy, improve public services, and enhance the quality of life for its citizens. Within the healthcare sector, Vision 2030 has already catalyzed a monumental shift toward a patient-centric framework. Key strategic objectives include a significant increase in the healthcare workforce and a substantial expansion of hospital capacity, crucial for accommodating the acute and sustained demands of mega-events like the World Cup. This transformation also emphasizes advanced health information technology, including the adoption of electronic health records and telemedicine services, laying a vital foundation for integrated care [2]. The commitment under Vision 2030 to create a vibrant society and a thriving economy is inherently linked to establishing a world-class healthcare system that is prepared for any public health challenge, especially those posed by hosting an event of this magnitude.

Beyond physical infrastructure, the true transformative innovation under Vision 2030 is its aggressive digital pivot within healthcare. The strategic integration of advanced digital systems, including artificial intelligence (AI)-driven diagnostics and expansive telemedicine networks, is actively dismantling traditional barriers to care accessibility, particularly in remote and underserved regions [3]. These reforms are not merely aspirational but demonstrably operational. To illustrate, Saudi Arabia has extensively invested in developing a robust digital health ecosystem that supports seamless data flow and remote patient management, crucial capabilities for any mass gathering. Recent mass gatherings such as the annual Hajj pilgrimage offer invaluable, albeit challenging, lessons for future event preparedness. During Hajj 2024, unprecedented temperatures reaching 51°C led to significant heat-related morbidity and mortality. This event starkly underscored the escalating impact of climate change on large-scale gatherings, emphasizing the critical need for sophisticated environmental adaptation strategies and real-time public health interventions [4,5]. The Hajj experience directly informs the World Cup preparations by highlighting the urgency of implementing dynamic heat management protocols and robust health surveillance systems. The Kingdom’s proactive investment in predictive analytics and mobile health (mHealth) platforms, which facilitate health-related communication via mobile devices, further aligns with global trends toward data-driven emergency medicine [3]. Lessons from Qatar’s 2022 World Cup, where rapid genomic sequencing successfully contained potential COVID-19 outbreaks among a global population, offer actionable frameworks for infectious disease surveillance [6]. Saudi Arabia must leverage its Vision 2030 momentum to pioneer a proactive rather than merely reactive model of emergency care for the World Cup, building on these experiences and global insights [5]. This proactive approach will integrate continuous risk assessment and predictive modelling to anticipate and mitigate health threats before they escalate, moving beyond traditional emergency response frameworks.

Pioneering a health-first approach: key principles and innovations for 2034

The 2034 FIFA World Cup offers a unique operational laboratory to advance several core principles in mass gathering health and sports medicine, thereby establishing a comprehensive, integrated emergency preparedness model that sets a new international benchmark.

Advanced Digital Health Integration and Interoperability for Seamless Care

The future of mass gathering medical response necessitates the widespread adoption of integrated digital health technologies. Wearable devices, capable of continuously monitoring vital signs such as heart rate, oxygen saturation, and body temperature, can provide real-time data to AI-powered triage systems, enabling early detection of distress and prioritized interventions [3]. Imagine a scenario where thousands of attendees’ aggregated biometric data, anonymously collected from wearables, could alert medical staff to potential widespread heat exhaustion, allowing for targeted interventions in specific areas. Drone-delivered medical supplies, including automated external defibrillators and critical medications, can significantly reduce response times in congested environments, improving patient outcomes in time-sensitive emergencies [7]. The integration of secure, interoperable health data systems, potentially leveraging advanced digital frameworks for health information exchange, is crucial for coordinated responses among multilingual, multinational care teams [2,8]. This would mean that a patient’s essential medical history and current status could be instantly and securely accessed by authorized medical personnel, regardless of where the incident occurred or the patient’s nationality, ensuring continuity of care and preventing medical errors. Such systems are vital for effective patient management during complex emergencies involving a diverse global population.

Robust Climate Resilience and Predictive Environmental Health Monitoring

With global temperatures consistently rising due to climate change, extreme heat poses an increasingly significant environmental threat to outdoor events like the World Cup [5]. The imperative for robust heat stress monitoring is underscored by studies conducted in Saudi Arabia, where industrial workers frequently experienced Wet Bulb Globe Temperature (WBGT) values exceeding recommended limits, leading to severe heat strain. WBGT is a widely recognized index that accounts for temperature, humidity, wind speed, and solar radiation, providing a more accurate assessment of heat stress than air temperature alone. Therefore, the widespread adoption of advanced WBGT monitoring systems, similar to those used by international sports federations, combined with Saudi Arabia’s planned deployment of numerous hydration and cooling stations, must become standard practice to safeguard the health of attendees [4,9]. This includes not only stationary cooling centers but also mobile cooling units and misting stations strategically placed in high-traffic areas, along with readily available electrolyte solutions. Furthermore, predictive environmental modelling, based on hyper-local weather data, can anticipate heat spikes hours or days in advance, allowing organizers to issue specific advisories, adjust event schedules, and deploy resources pre-emptively. Proactive heat acclimatization guidance for athletes and spectators, alongside real-time, multilingual public health messaging through dedicated mobile applications and stadium screens, will be vital to encourage self-care and early recognition of heat-related illness [5]. These measures are critical for preventing mass casualties from heatstroke, as tragically demonstrated during Hajj 2024.

Ensuring Health Equity in Emergency Care Access and Delivery

The ultimate success of the World Cup, from a public health and ethical perspective, hinges on ensuring equitable access to care for all participants, irrespective of their socioeconomic status, nationality, or employment [8]. Saudi Arabia’s Vision 2030 explicitly emphasizes “patient-centric care,” but this principle must extend unequivocally to vulnerable populations, including migrant workers and low-income attendees [2]. The broader social impacts of mega-events, including potential inequities, require careful consideration to ensure benefits are widely distributed and burdens minimized [1]. This demands transparent labor practices, fair wages, and comprehensive health insurance for all workers involved in the World Cup infrastructure, from construction to event operations. Proactive measures, such as easily accessible and affordable healthcare services, clear communication in multiple languages regarding available medical services, including clear signage and multilingual staff at medical points, and dedicated medical support for specific worker populations, are non-negotiable for true health equity. Ensuring cultural competency among medical staff and providing translation services will further enhance the patient experience for a truly global audience, guaranteeing that no individual’s health is compromised due to language barriers or lack of resources.

Leveraging MedTech for Proactive Emergency Preparedness and Response

By treating the 2034 World Cup as a proving ground, Saudi Arabia can pioneer a cutting-edge emergency preparedness model that seamlessly integrates state-of-the-art MedTech innovations. This includes sophisticated AI-driven surveillance systems employing machine learning algorithms to forecast disease outbreaks by analyzing complex datasets such as travel patterns, environmental data, syndromic surveillance from clinics, and even real-time public health data from various points of entry. This enables proactive resource allocation and targeted interventions [10]. Such systems can identify nascent disease clusters and predict their spread, allowing for immediate public health responses such as targeted awareness campaigns or rapid diagnostic testing. Furthermore, the extensive deployment of robotics and autonomous systems can dramatically enhance response capabilities. This could involve medical drones for rapid delivery of critical supplies to remote or congested areas [6] and robotic exoskeletons to assist mobility-impaired spectators, ensuring their safe navigation within venues or during evacuations. Automated disinfection robots could also maintain hygiene standards in high-traffic areas, further mitigating infectious disease transmission risks, contributing to a cleaner and safer environment for all attendees. Integrating these technologies within a comprehensive health infrastructure framework can create a highly responsive and adaptive medical environment [5].

A call to action for emergency medicine professionals and policymakers

The 2034 World Cup presents a compelling global health imperative, demanding concerted engagement from all stakeholders in cross-sector research and development. This includes active partnerships with international health organizations, such as the World Health Organization, and sports federations, such as FIFA, to validate critical strategies for heat mitigation, crowd management, and infectious disease surveillance in arid climates, drawing invaluable lessons from Saudi Arabia’s extensive experiences [4,5]. Such collaborations are essential for sharing best practices, developing standardized protocols, and ensuring that the health and safety measures implemented are globally recognized and effective. It further necessitates prioritizing ethical innovation, ensuring that the integration of AI and robotics serves to enhance, rather than supplant, human clinical judgment and compassion during emergencies [8]. The focus must remain on augmenting human capabilities, not replacing them, fostering trust in technological solutions while upholding the core values of medical care. Building robust surge capacity within the healthcare system is paramount, reinforced through international Emergency Medical Services partnerships, joint training exercises, and cross-border health risk assessments to ensure a truly resilient and globally responsive emergency response system for the World Cup and future mass gatherings [2,5]. This includes establishing clear lines of communication, pre-defined emergency protocols, and regular simulation drills involving all relevant agencies, both national and international, to ensure a seamless and efficient response to any medical incident.

Finally, it can be concluded that Saudi Arabia’s hosting of the 2034 FIFA World Cup is poised to become a landmark event in the evolution of emergency medicine innovations for mass gatherings. By judiciously harnessing the digital transformation momentum of Vision 2030, integrating global trends in prehospital and disaster medicine, and rigorously applying invaluable lessons gleaned from the Hajj pilgrimage, the Kingdom can establish an unprecedented new standard for emergency preparedness. This forward-looking standard will unequivocally prioritize cutting-edge innovation, ranging from advanced real-time monitoring and AI-powered triage systems to sophisticated, secure data-sharing platforms, without ever compromising on the fundamental principle of equity in healthcare access and delivery. The proactive integration of technology, coupled with a deep understanding of unique environmental challenges and a commitment to inclusive care, will be the hallmarks of this new standard. The stakes extend far beyond the realm of football; this is a truly unprecedented opportunity to demonstrate that a comprehensive “health-first” approach to mega-events, meticulously driven by pioneering emergency medical solutions, is not merely possible but an absolute imperative for the future of global public health and mass gathering safety. The legacy of the 2034 World Cup could well be a global blueprint for managing the health risks of large-scale events in an increasingly complex and interconnected world.
